# Core outcome set and measures of chest health in children and young people with cerebral palsy in the community setting: the CHESTI study protocol

**DOI:** 10.1136/bmjopen-2025-105309

**Published:** 2025-08-11

**Authors:** Rachel Knight Lozano, Christopher Morris, Harriet Shannon, Kayleigh Bell, Hugh Malyon, Julia Melluish, Jos Latour, CHESTI-study steering group, Morag Andrews

**Affiliations:** 1 University of Plymouth, Plymouth, UK; 2 University of Exeter Medical School, Exeter, UK; 3 University College London Great Ormond Street Institute of Child Health, London, UK; 4 Public Contributor, Exeter, UK; 5 Public Contributor, Torbay, UK; 6 Public Contributor, Devon, UK

**Keywords:** Child, Community child health, RESPIRATORY MEDICINE (see Thoracic Medicine), NEUROLOGY, Treatment Outcome, Patient Reported Outcome Measures

## Abstract

**Abstract:**

**Introduction:**

Poor chest health is the leading cause of early mortality in children with cerebral palsy (CP). It is also the most common reason to seek healthcare, accruing significant costs and reducing quality-of-life for children and families. Clinical trials examining chest health interventions in CP are characterised by inconsistent outcome measures, limiting the capacity for evidence synthesis to inform clinical application. The study aims to develop a core outcome set (COS) and related measurement instruments to assess, monitor and evaluate chest health in children with CP, both in research and routine clinical practice. The COS will reflect the views of children, young people, parent/carers, clinicians and researchers, emphasising under-represented groups in research and those at risk of poorer chest health.

**Methods and analysis:**

A 3-phase methodology will be conducted in line with the Core Outcome Measures in Effectiveness Trials (COMET) Initiative. (1) Candidate outcomes will be identified through a qualitative evidence synthesis and interviews with key stakeholders. Findings will be mapped to COMET-taxonomy, generating a list of candidate outcomes. (2) An international e-Delphi survey will invite stakeholders to rate the importance of each outcome, followed by a consensus meeting to ratify the COS. (3) A structured review, guided by health measurement taxonomy, will evaluate relevant instruments, with a final meeting to agree on recommended measures for each COS domain.

**Ethics and dissemination:**

Ethical approval was provided by the University of Plymouth Research Ethics Committee for the qualitative interview study (ID5116), e-Delphi study and consensus meeting (ID5636). Study findings will be published open access in a peer-reviewed journal and presented at relevant national and international conferences.

**Study registration:**

COMET registration: 2590 (https://www.comet-initiative.org/Studies/Details/2590)

**PROSPERO registration number:**

CRD42024562735.

**STRENGTHS AND LIMITATIONS OF THIS STUDY**

- Proposed core outcome set (COS) methods are rigorously guided by Core Outcome Measures in Effectiveness Trials guidelines.
- COS development will be informed by views of children, young people, parent/carers, clinicians and researchers, emphasising under-represented groups in research and those at risk of poorer chest health.
- COS development and application extends to implementation in research trials and routine clinical practice, aligning through the healthcare system.
- Despite efforts for international e-Delphi reach, the COS may be limited by potential imbalance between national and international participants.
- In progressing to identification of outcome measurement instruments, we anticipate that there may be no suitable measurements for some domains of the COS.

## Background

 Cerebral palsy (CP) is the most common physical disability in early childhood, affecting 30 000 children in the UK and 17 million people globally.^1^ CP is a lifelong condition caused by permanent damage to the developing brain. This can affect a person’s ability to move, and also to swallow, breathe, cough and clear their lungs effectively, leading to poor chest health and recurrent illnesses.^2 3^ For five decades, chest-related illness has been the leading cause of early death in children with CP.^2 4^ In the UK, an estimated 250 children with CP die each year, of whom 51% result from poor chest health.^5^ Mortality rates are higher in low and middle-income countries, rising from 5.3/1000^5^ to 19.5/1000.^6^ Chest-related illness is also a major reason for children with CP to attend hospital, accruing significant healthcare costs, reducing participation and impacting on quality-of-life for the child and their family.^7^

CP presents a distinct scope of coexisting impairments that predispose the child to chest-related morbidity, many of which are modifiable.^8^,^10^ This presents an opportunity to prevent recurrent illnesses and worsening chest morbidity through proactive assessment, monitoring and timely management of chest health in routine clinical practice.^3^ Such preventative strategies are recognised in paediatric priority setting partnerships,^11^ broader healthcare initiatives^12^ and National Institute of Clinical Excellence guidelines,^13^ informing standards of care for other childhood-onset conditions, such as cystic fibrosis^14^ and muscular dystrophy.^15^ However, these standards of care rely on ‘gold-standard’ measures of lung function, such as spirometry, which can be challenging to replicate in children with CP. This presents a major barrier to implementing proactive care in those at higher risk of recurrent chest illnesses, including children with severe motor impairments and/or learning difficulties,^8 10^ contributing to ongoing health and healthcare disparities in this population.

Chest health outcomes are also measured in research and routine clinical practice to evaluate the impact of interventions on a person’s health or quality-of-life.^16^ Pharmacological and non-pharmacological treatments are widely prescribed by healthcare professionals to manage symptoms, reduce illness burden, lower emergency hospital visits and improve quality-of-life for children with CP.^17 18^ Yet, our recent scoping review mapped 76 different chest health measurements across 78 studies worldwide, concluding no consensus of what or how to measure this concept in children with CP.^19^ Findings resonate with a recent consensus study^9^ and two existing intervention systematic reviews,^20 21^ agreeing the current landscape of research is characterised by low quality methodology and inconsistent measures. This presents a significant barrier to informing evidence-based treatment and standards of care in children with CP.

A core outcome set (COS) is a standardised set of agreed outcomes that should be measured and reported within a specific area of health or healthcare.^22^ Application of COS in clinical research trials enhances relevance of studies, reduces waste and minimises reporting bias. Furthermore, it addresses issues of inconsistent measures and enables pooling of similar findings across multiple studies to inform evidence-based treatment decisions.^16 23^ Recently, COS development and application have moved beyond research trials, into routine clinical practice, aligning through the healthcare system, with novel examples featuring in Core Outcome Measures in Effectiveness Trials (COMET)^24^ and The International Consortium of Health Outcome Measurement.^25^ Applying COS in clinical practice facilitates early assessment and monitoring of chest health in children with CP, facilitating timely interventions that help to reduce illness and associated hospital care.^8^ Additionally, it supports efficient routine data collection, which can inform clinical trials, audit and quality improvement efforts, bridging the gap between research and real-world practice. Moreover, development of a COS in partnership with lived experience experts, such as children, young people and carers, aligns with value-based healthcare and commissioning. This ensures that health resources are directed to health outcomes that matter most to patients, maximising impact and benefit.^16^

Despite the need and potential benefits to research and routine clinical practice, no COS currently exists to assess, monitor or evaluate chest health in children with CP. The aim of this study is to develop and agree a COS and measurement instruments to assess, monitor and evaluate chest health in children with CP, in research and routine clinical care. This will be informed by lived experience experts, clinicians and researchers internationally. Specifically, we will (1) identify candidate outcomes of chest health in children with CP; (2) determine which chest health outcomes are most important to key stakeholders and (3) recommend best available outcome measure instruments (OMIs) for each agreed core outcome domain.

## Methods and analysis

### COS overview

**Figure 1:**
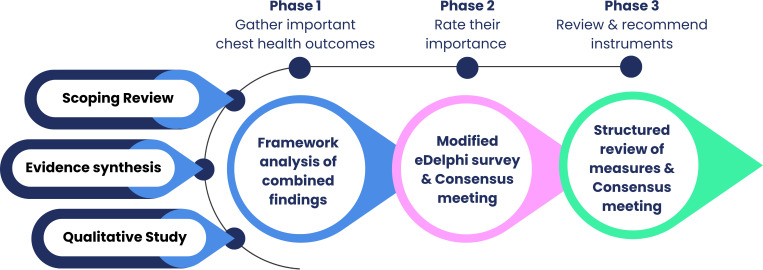
Overview of COS methods. COS, core outcome set.

The core outcome set and measures of chest health in children and young people with cerebral palsy in the community setting (CHESTI) study was registered with the COMET database in March 2023 (ID2590 http://www.comet-initiative.org/Studies/Details/2590). This three-phase study design (figure 1) incorporates development and agreement of COS domains and associated core OMI, as follows:

- *Phase 1*: candidate outcomes will be identified through an evidence synthesis and qualitative interviews with key stakeholders, including children and young people, parent/carers and clinicians.
- *Phase 2*: candidate outcomes will be rated for importance via an international e-Delphi study, by children and young people, parent/carers, clinicians and researchers. Outcome domains reaching a threshold for consensus will inform the COS, which will be ratified in a consensus meeting.
- *Phase 3*: measurement instruments will be identified and evaluated for each core outcome, to assess reliability, validity, responsiveness, interpretability, appropriateness, precision, acceptability and feasibility. OMIs will be agreed for recommendation in a final consensus meeting with key stakeholders.

The COS design is underpinned by recommendations from the COMET Handbook^22^ and COS-STAndards for Development (COS-STAD).^26^ For transparency and completeness, the protocol is reported in line with the Core Outcome Set Standardised Protocol Items^27^ and the final COS will be reported using the COS-Standards for Reporting.^28^

### Scope

The COS scope (table 1) is intended as a minimum international standard of important outcomes and associated measures that can be used in both research and routine clinical practice. The population will include all children aged 1–18 years with a health condition of CP, including those with multiple comorbidities. The scope will consider outcomes implemented in a community setting, evaluating the effect of any intervention where the aim is to improve chest health, or the progression of chest health if no intervention is given. The scope has been codeveloped with public contributors, emphasising its relevance and inclusivity for those at risk of poorer chest health outcomes.^29^

**Table 1:** COS scope based on the Core Outcome Set-STAndards for Development

Scope concept	Definition
Population	Children aged 1–18 years have been selected to maximise patient impact and benefit, with lower age-limit representing those with increased healthcare use,^5^ while the upper age-limit encompasses young people at increased risk of chest morbidity.^55^
Health condition	CP was selected as an exemplar of neurodisability, informed by its prevalence and well-researched association with chest-related morbidity and mortality.^5^ This scope emphasises a wide spectrum of CP, including children and young people with comorbidities, severe motor and learning disability, due to their poorer chest health outcomes and under-representation in current literature.^13^
Intervention	Outcomes are defined as the effect of any intervention where the aim is to improve chest health, or the progression of chest health if no intervention is given.^16^
Setting	The scope will consider outcomes that are feasible to apply in a community settings, underpinned by proactive models of care driven by James Lind Alliance priorities^11^ and healthcare agenda.^12^

COS, core outcome set; CP, cerebral palsy.

### Conceptual framework

The COMET taxonomy has been selected as a comprehensive framework to develop the COS items, aligning with its primary purpose to inform classifications of health outcome.^30^ It sets out multifaceted categories underpinned by conceptual and empirical work, which reflect the complexity of the health condition of interest.

### Stakeholders

To maximise inclusive patient benefit and impact, key stakeholder views will inform each phase, identifying candidate outcomes in phase 1, rating the importance of each outcome in phase 2 and contributing to OMI recommendations in phase 3. Stakeholders include professionals involved in relevant research, health, education and social care, children and young people with CP and their parents/carers (table 2). Efforts will be made to engage groups at risk of poorer chest health outcomes, including families of ethnic minority or low socioeconomic status, the historically marginalised and those caring for children with multiple comorbidity or learning disability.^5 8 29^

**Table 2:** Stakeholder eligibility

Stakeholder		Definition
Lived experience experts	Patients	Children and young people aged from 8 to 25 years old. Diagnosed with cerebral palsy (CP). Have experience of previous or ongoing chest health issues.
	Parent/carer	Family or carer of a child or young person (CYP) diagnosed with CP. Have experience of caring for their child with previous or ongoing chest health issues.
Health, social and educational professionals		Allied health (physiotherapists, dieticians, speech and language therapists, etc), nurses and medical professionals. At least 2 years of clinical experience in CP and/or chest health working predominantly in the community setting (home, school, outpatients).
Academics and researchers		Triallists, systematic reviewers or clinical academics working in the field of CP and/or chest health in the wider field of neurodisability.

### Patient and public involvement

Parent/carers and young people with lived experience have codesigned, reviewed and refined each phase of this protocol, leading on accessible terminology and study information, and contributing to the COS scope and participant eligibility. Referred to as public contributors, they have also emphasised the importance of OMI acceptability in the final phase, to reflect comorbidity and learning difficulties in children with CP, striving to include populations previously underserved in research. Planned public contributor activity within each phase of the COS development is presented in table 3 and will be reported using the Guidance for Reporting on Involvement of Patients and Public short-form checklist.^31^

**Table 3:** Public contributor activity

Phase	Activity
Phase 1	Codevelopment of recruitment strategy and topic guide for semi-structured interviews. Codevelopment of public-facing information to support sensitive, diverse recruitment. Verification of findings and refinement of potential outcomes for the e-Delphi survey.
Phase 2	Codevelopment of an e-Delphi survey design. Sharing of plain English findings between each Delphi round. Codevelopment of two animated videos to support participation and dissemination.
Phase 3	Verification of content validity findings for itemised outcome measure instruments.
Dissemination	Sharing of findings and direction of dissemination pathways for each research phase.

### Steering committee

A group of 8–10 stakeholders will meet two times yearly to influence research strategy decisions, referred to as the CHESTI-steering group. The group includes an independent chair, an equality and diversity representative, members of the research team and collaborating partners, two public contributors and experts in clinical or research delivery of chest health and neurodisability.

## Phase 1: identifying candidate outcomes

Early implementation of qualitative methods emphasises stakeholder-informed outcome domains, generating new understandings and setting a meaningful agenda for COS development and application.^32^ Phase 1 aims to identify stakeholder-informed candidate outcome domains of chest health through a UK-based interview study and an international qualitative evidence synthesis. Findings will be combined to generate a list of outcomes considered important to stakeholders, informing survey items for Phase 2.

### Qualitative evidence synthesis

#### Methods

Building on our previous scoping review,^19^ a qualitative evidence synthesis (PROSPERO. CRD42024562735) will be conducted to generate an international catalogue of candidate outcomes, underpinned by differing global perspectives, experiences and context.^22^ The review aims to identify, analyse and synthesise views of chest health in CP, according to lived experience experts, carers and professionals worldwide. Searches in Cochrane Library and PROSPERO confirm no existing reviews within this research topic of interest.

#### Searches and sources

Systematic searches will be conducted with support from a senior information specialist. Search terms will be codeveloped with the CHESTI study steering group. Databases will include MEDLINE (Ovid), EMBASE (Ovid), Allied and Complementary Medicine Database (EBSCOhost), Cumulative Index of Nursing and Allied Health Literature (EBSCOhost), PsycINFO (ProQuest) and Scopus, with additional grey literature searches to minimise publication bias. Retrieved articles will be stored in Endnote and transferred to JBI SUMARI, to support a transparent audit trail for selection of papers.

#### Study selection

A two-stage screening process will be undertaken by independent reviewers determining eligibility at title and abstract, and at full text, involving a third member to resolve discrepancies. Studies will be eligible if they (1) explored experiences of chest health, associated illnesses and health concepts, in children (mean age <18 years) with CP; (2) from the perspectives of children and young people, their parent/carer or healthcare professionals; (3) implemented qualitative or mixed methods in which qualitative data could be extracted. Studies of any geographical origin and language will be considered. Opinion pieces, editorials, reviews and quantitative studies will be excluded. The JBI Critical Appraisal Checklist will be used for quality assessment of the included studies.^33^ To avoid omitting rich and relevant data, quality assessment will inform discussion but will not be a threshold for exclusion.^34^

#### Data synthesis

Data will be synthesised using meta-aggregation, in accordance with the JBI approach^35^ as follows: (1) findings from included studies will be extracted verbatim, alongside illustrative primary data. A level of credibility will be allocated to each finding, defined as ‘unequivocal’, ‘credible’ or ‘unsupported’. Unsupported data will not progress to analysis; (2) extracted findings will be categorised based on key concepts arising from similar findings; (3) categories of similar meaning will be grouped to contribute synthesised findings expressed as indicatory statements. Each step will be conducted with a team of three independent researchers and reviewed by the wider research team. A consensus of extracted findings, level of credibility, categories and synthesised findings will be reached through discussion, and refined, with a third reviewer to resolve disagreement.

### Qualitative study

#### Methods

Alongside the evidence synthesis, a primary qualitative study will be conducted to explore experiences of chest health, associated illness and outcomes in children with CP, sought through semistructured interviews with children and young people, parent/carers and professionals. To maximise inclusive patient benefit and impact, there is particular emphasis on recruitment of underserved research cohorts and those at risk of poorer chest health, including families identifying as ethnic minority or low socioeconomic status and children with diverse severity of CP.^5 8 29^ This study will be reported in line with the Consolidated Criteria for Reporting Qualitative.^36^

#### Participants

Participant eligibility was codeveloped with public contributors and includes (1) children with CP and a lived experience of a chest illness;^37^ (2) parent/carers of children with CP and a lived experienced of a chest illness; (3) health, social care and educational professionals with relevant clinical experience. Participants will be able to provide consent or have access to a parent able to consent on their behalf. Access to interpreters, familiar communication partners and/or the use of augmented communication strategies such as Talking Mats will be offered to support diverse communication needs. Where a child is unable to share their views due to a severe learning or communication need, the carer will be invited to interview as proxy to ensure children with severe impairments are represented.

#### Procedures

A purposive sampling strategy will be implemented to recruit up to 30 stakeholders, representing (1) children and young people with CP; (2) parent/carers; (3) and health, social care and educational professionals. A sampling matrix, stratified by age and severity of CP, will be implemented, to reflect factors associated with healthcare usage and chest illness.^5 8^ Relevant professionals will be stratified by discipline to reflect the different aspects of care delivery. Children and families will be invited through regional study champions, with specialist knowledge of relevant local charities, special schools, support groups and private organisations. Accessible study information will be codeveloped with collaborating partners and public contributors and offered in alternative languages. Health, social, educational and research professionals will be invited through gatekeepers of professional bodies, specialist interest organisations and research networks.

#### Data collection

Semistructured interviews will be conducted by the principal investigator (PI) (RKL), trained in qualitative research methods, using participant-preferred face-to-face, online or telephone methods, to reduce participant burden and reach a wide geographical representation. Collaborating partners and public contributors will support codevelopment, pilot and refinement of a relevant, culturally sensitive topic guide to be implemented at interview.

#### Data analysis

Each interview will be audio-recorded, transcribed verbatim and uploaded to NVivo V.12, supported by observational field notes. Inductive framework analysis will be used to support synthesis of qualitative data, built from data-driven emergent concepts and themes.^38^ A second researcher, trained in qualitative research methods, will review each stage to minimise researcher bias. Findings from each phase 1 study and a previously published scoping review^19^ will undergo secondary framework analysis, indexed to the COMET-taxonomy,^30 38^ generating a list of outcome domains. This will be reviewed and refined in partnership with public contributors, research partners and the steering group, to finalise a list of candidate chest health outcomes for the next phase.

## Phase 2: agreeing important outcomes

The aim of phase 2 is to seek consensus on ‘which’ candidate outcome domains of chest health are most important, through the views of relevant expert stakeholders. This will be determined through an international e-Delphi study and final consensus meeting.

### Methods

An international e-Delphi survey method will be implemented in line with COMET recommendations.^22^ This will be executed using general data protection regulation approved software, maintaining anonymity and minimising unfavourable group dynamics.^22^ It also increases potential for global reach, reduces participant burden, while supporting diverse representation of stakeholders. The e-Delphi survey will be codesigned and piloted with public contributors, collaborating partners and COMET representatives, to ensure the process is valid and easily understood.

### Participants

To support COS application in research and routine clinical practice, public contributors suggested that participants ‘must have experience working closely with children with cerebral palsies and have knowledge of their day-to-day needs’. Participants will represent three panels (1) lived experience experts, defined as children and young people with CP and their parent/carers; (2) health, social and educational professionals; (3) academics and researchers. In the absence of an evidence-based guideline for optimal sample size, 15–20 participants will be recruited across each of the three panels, based on the minimum sample size for high replicability.^22 39^ This is comparable to CP-based COS studies, acknowledging that only a subset of the CP population may be affected by chest health problems.^40^

### Procedures

Participants will be informed of the e-Delphi study purpose, rationale and process through a participant information sheet and bespoke CHESTI-study animated video, with subtitles. This information will be made available to facilitate informed consent and establish expectations, purpose and potential impact for participation.^22^ Information will be adapted to meet the unique needs of each stakeholder panel and assessed for accessibility by available software, public contributors and collaborating partners.^22 41^ Information will aim to be translated into six languages, defined by the WHO, to provide culturally sensitive written study materials. Lived experience experts will be invited through gatekeepers of international school networks, parent/carer organisations, charities and CP-focused registries. Academics and researchers will be invited through author publications and higher education networks, while health, social and educational professionals will be invited via gatekeepers of professional bodies, specialist interest groups, and other global paediatric professional organisations.

### Data collection

Up to three e-Delphi survey rounds are proposed to converge views and support an ‘iteration effect’,^42^ without increasing risk of attrition bias.^22^ Participants will be asked to rate the importance of each outcome domain using a Likert rating scale.^43^ There will be an ‘unable to score’ option, and opportunity to add comments, generating contextual understanding, and to suggest any outcome domains they think are missing. Feedback will be given between rounds, providing the opportunity for panellists to modify their rating with knowledge of other survey responses.

### Data analysis

After each round, ratings will be analysed within panels and between panels.^43^ Open text data will be extracted verbatim and thematic analysed by the PI (RKL), both within panel and between panel.^44^ Initial codes and themes will be reviewed by the wider supervisor team for quality assurance. Free text comments, level of importance (expressed as median) and level of agreement (expressed as a percentage) will be summarised and fed back to respondents, sharing an understanding of wider opinions and scores, facilitating ‘vicarious thinking’ and reflection between rounds.^45 46^ A threshold for ‘critically important’ items has been defined *a priori* in line with COS-STAD^26^ (table 4). Threshold for consensus will employ the 70%/15% rule, in which 70% of respondents rate an outcome critically important, and 90% from any single panel group rate an outcome critically important.^22 47^

**Table 4:** Definition of consensus^47^

	Consensus ‘In’	Consensus ‘Out’	No consensus
Between-panel	≥70% of responses consider an item ‘critically important’ and ≤15% of responses consider an item ‘not that important’	≥70% of responses consider an item ‘not that important’ and ≤15% of responses consider an item ‘critically important’	Anything else
Within-panel	>90% of responses consider an item ‘critically important’	>90% of responses consider an item ‘not that important’	Anything else

### Final consensus meeting

A multistakeholder consensus meeting will be held within 3 months of the final round, to ratify agreement of outcome domains derived from survey findings.^22^ Participants will include 4–5 e-Delphi respondents from each panel and representatives from the CHESTI-study steering group. This will be held virtually to minimise participant burden and facilitate wide geographical reach. Inclusion of young people with CP who may experience communication difficulties and/or a learning disability will be supported through familiar communicators and online platform accessibility functions. The meeting will be structured using an agenda, agreed by the CHESTI steering group. This will include ratification of ‘consensus In’ items across panels, ‘consensus In’ items between panels, and ‘borderline consensus’ items across or within panels. Thresholds for discussion have been defined *a priori* to reduce risk of researcher bias (table 5).^26^ We will agree a minimum COS for chest health in children and young people with CP, informed through inclusive and participatory stakeholder views.

**Table 5:** Definition of ‘borderline’ items for final consensus meeting

Scenario for discussion of ‘borderline consensus’		Defined as
Between-panel ‘borderline’	Where two of three panels *do* meet within-panel ‘consensus In’, but *do not* meet between-panel ‘consensus In’	>70–89% score critically important *and *<15% score not that important, in two of three panels
Within-panel ‘borderline’	Where one panel *does not* meet within-panel ‘consensus In’ *but* is considered *borderline*.	>85–89% score critically important in one single panel
Between-panel ‘borderline’	Where all three panels *do not* meet criteria between-panel ‘consensus In’ (>70%) *but* all are considered *borderline*.	>65–69% score critically important and <15% score not that important in all three panels

## Phase 3: health measurement review

The aim of phase 3 is to recommend best available OMIs for each agreed core outcome domain of chest health in children and young people with CP. This will be determined through a structured health measurement review, providing an overview of relevant existing OMIs and their measurement properties, to inform agreement of the best available OMIs for each agreed core outcome. Public contributors and collaborating partners emphasise the selection of feasible ways to measure items of the COS, to progress its application into research and routine clinical practice.^19^

### Methods

Evidence synthesis methodology will be employed to systematically identify, evaluate and inform recommended OMIs. Selection will be determined by (1) OMI relevance to COS domains, specified population and context of interest; (2) quality of measurement properties and their underpinning studies; (3) feasibility and acceptability based on view of stakeholders.^48^

### Identifying candidate OMIs

Scoping review searches^19^ will be updated to identify new candidate OMIs since 2023, alongside existing reviews of OMIs relevant to the agreed COS. All candidate OMIs will be shared with public contributors and collaborating partners to verify relevance to the COS scope and agreed domains, while also detecting additional OMIs used in practice. Any itemised candidate OMIs will undergo assessment of content validity, drawing on established guidance to examine comprehensiveness and comprehensibility.^49^ Two independent reviewers will extract and map candidate OMI items to COS domains. Discrepancies will be resolved through comparison and discussion, with support of a third reviewer. Public contributors will verify this process to ensure authentic application.

### Review of measurement properties

Candidate OMIs demonstrating content validity will undergo a review of published psychometric properties. We will evaluate measurement properties, drawing on health measurement taxonomy to evaluate reliability, validity, responsiveness, interpretability, appropriateness, precision, acceptability and feasibility, with international consideration of language.^50 51^ Findings will be summarised and presented in an accessible Table of OMI properties for each COS domain.

### Final consensus meeting

A group of 8–12 stakeholders, including lived experience experts and relevant professionals, will participate in an online multistakeholder consensus meeting. Candidate OMIs for each COS domain will be presented and discussed to support the recommendation of authentic and acceptable OMIs. These OMIs will be recommended alongside the COS, to assess, monitor or evaluate chest health in children and young people with CP in research and routine clinical practice. Where a COS domain cannot recommend an OMI, validation research or OMI development will be proposed as future research.

## Ethics and dissemination

Ethical approval has been granted by the University of Plymouth (ID 5116; ID 5636). Dissemination of the COS and associated OMIs will be facilitated through relevant professional clinical, education and research networks, and organisations or charities that represent health professionals, families of children and young people with CP. Findings will be shared in a one-page plain English summary, an infographic and an animation video, with subtitles. Additional correspondence with journal editors and authors publishing in this field will be sought to support uptake in research. Dissemination workshops with clinicians will be held to support uptake in routine clinical practice. Findings will be submitted for publication in peer-reviewed, open access journals and presented at national and international conferences within respiratory and childhood neurodisability.

## Discussion

To our knowledge, no COS currently exists to assess, monitor or evaluate chest health in children and young people with CP. The COMET database features existing COS in primary^52^ and secondary^53^ respiratory diseases but recommend measures that are not widely replicable in child or young person (CYP) with CP. The registry also includes a broad COS in CP, considered to be insensitive to neurorespiratory-related impairments.^40^ This COS protocol employs a well-established and widely used design developed by the COMET Initiative.^22^ It moves beyond traditional COS boundaries, developing and applying a COS into both research and routine clinical practice. Such application is of growing interest, aligning outcomes through the healthcare system and bridging the gap between research and practice.^24^

Our recent scoping review mapped existing published outcome domains and associated OMIs to evaluate chest health in children and young people with CP.^19^ Yet, published clinical research may not capture or report outcome domains considered most important to children and their families.^54^ Furthermore, this review found almost 60% of studies excluded participants with severe motor impairment and/or a learning disability, underserving those at highest risk of chest-related morbidity. Involving lived experience experts in COS development has become common practice to ensure the relevance of the proposed COS to all stakeholders. In this COS protocol, young people with CP, their parents/carers, clinicians and researchers have been involved in the protocol development and will have the opportunity to continue engagement through public contributor activity and steering committee participation, and also as research participants in the interview, e-Delphi and final consensus meeting processes.

The protocol has some limitations. It is anticipated that this COS will be developed for international reach. However, the authors acknowledge that potential imbalance between national and international participants may limit global application. Despite efforts to mitigate this through translating study information and diverse recruitment strategies, the study also risks under-representing populations with reduced health literacy or digital capability or capacity to complete the e-Delphi survey. Finally, the COS aims to progress to recommended OMIs to assess, monitor or evaluate chest health in CYP with CP. However, we anticipate that for some core outcomes, we may not be able to recommend a suitable OMI. This will inform the need for future research or OMI development opportunities to continue the momentum to improve chest health for CYP with CP.
